# Anti-HSV-2 activity of *Terminalia chebula* Retz extract and its constituents, chebulagic and chebulinic acids

**DOI:** 10.1186/s12906-017-1620-8

**Published:** 2017-02-14

**Authors:** Ajay Kesharwani, Suja Kizhiyedath Polachira, Reshmi Nair, Aakanksha Agarwal, Nripendra Nath Mishra, Satish Kumar Gupta

**Affiliations:** 1 Reproductive Cell Biology Laboratory, National Institute of Immunology, Aruna Asaf Ali Marg, New Delhi, 110 067 India; 2 Corporate R & D Centre, HLL Lifecare Limited, Akkulum, Thiruvananthapuram, Kerala 695 017 India; 3 Present Address: Anatomy and Cell Biology, Medical Faculty, Saarland University, Homburg, Saar Germany

**Keywords:** Anti-HSV-2 activity, Attachment assay, Penetration assay, Post-infection plaque reduction assay, *Terminalia chebula*

## Abstract

**Background:**

Development of new and effective therapeutics for sexually transmitted herpes simplex virus-2 (HSV-2) infection is important from public health perspective. With an aim to identify natural products from medicinal plants, in the present study, the potential of *Terminalia chebula* Retz was investigated for its activity against HSV-2.

**Methods:**

Fruits of *Terminalia chebula* Retz were used to prepare 50% ethanolic extract. In addition, chebulagic acid and chebulinic acid both purified from *T. chebula* were also used. The extract as well as purified compounds were first used to determine their in vitro cytotoxicity on Vero cells by MTT assay. *T. chebula* extract, chebulagic acid, chebulinic acid along with acyclovir were subsequently assessed for direct anti-viral activity, and their ability to inhibit attachment and penetration of HSV-2 to the Vero cells. In addition, their anti-HSV-2 activity was also determined by in vitro post-infection plaque reduction assay.

**Results:**

Cytotoxicity assay using Vero cells revealed CC_50_ = 409.71 ± 47.70 μg/ml for the extract whereas chebulagic acid and chebulinic acid showed more than 95% cell viability up to 200 μg/ml. The extract from *T. chebula* (IC_50_ = 0.01 ± 0.0002 μg/ml), chebulagic (IC_50_ = 1.41 ± 0.51 μg/ml) and chebulinic acids (IC_50_ = 0.06 ± 0.002 μg/ml) showed dose dependent potent in vitro direct anti-viral activity against HSV-2. These also effectively prevented the attachment as well as penetration of the HSV-2 to Vero cells. In comparison, acyclovir showed poor direct anti-viral activity and failed to significantly (*p* > 0.05) prevent the attachment as well as penetration of HSV-2 to Vero cells when tested upto 50 μg/ml. However, in post-infection plaque reduction assay, *T. chebula* extract, chebulagic and chebulinic acids showed IC_50_ values of 50.06 ± 6.12, 31.84 ± 2.64, and 8.69 ± 2.09 μg/ml, respectively, which were much lower than acyclovir (71.80 ± 19.95 ng/ml).

**Conclusions:**

The results presented herein suggest that *T. chebula* extract, chebulagic and chebulinic acids have higher direct antiviral activity against HSV-2 and efficacy to inhibit virus attachment and penetration to the host cells as compared to acyclovir. However, acyclovir is more potent to inhibit post-infection virus replication. Hence, *T. chebula* may be a useful candidate for developing alternative therapy for prevention of sexually transmitted HSV-2 infection.

**Graphical abstract:**

ᅟ

## Background

Herpes simplex virus-2 (HSV-2) is a double stranded DNA virus belonging to the family *Herpesviridae*. It causes genital herpes, contributing to the risk of HIV infection through sexual route [1, 2]. HSV-2 is sexually transmitted virus and infect 500 million people worldwide and 20–23 million new infections are reported annually [3], http://www.who.int/mediacentre/factsheets/fs400/en/#hsv2. Since virus is an intracellular parasite in neural ganglia, it is difficult to completely eliminate it [1, 4]. Nucleoside derivatives such as acyclovir, valaciclovir, famciclovir and cidafovir have been widely used for the treatment of HSV infection [5]. In addition to high cost, treatment with acyclovir and other related drugs is also associated with the emergence of drug resistant strains [6], which is a major hurdle in the immunocompromised individuals, co-infected with other opportunistic Sexually Transmitted Infections (STIs) such as HIV-1 [7, 8].

Natural products from medicinal plants have been an important source of new biologically effective compounds exhibiting different modes of action against viral infection [9–11]. *Terminalia chebula* Retz. (*T. chebula*) is an evergreen flowering tree of the *Combretaceae* family and extensively used in traditional medicine. Pharmacological studies revealed the inhibitory activity of *T. chebula* on viral infections, such as human immunodeficiency virus type 1 (HIV-1), herpes simplex virus-1 (HSV-1), cytomegalovirus (CMV) and influenza [12–16]. In addition, the extract from *T. chebula* also has anti-bacterial, anti-fungal, anti-carcinogenic, anti-oxidant, anti-diabetic and anti-aging activities [17]. *T. chebula* has various phytoconstituents like tannins, flavonoids, sterols, amino acids, fructose, resins and fixed oil etc. However, tannins such as chebulinic acid, chebulagic acid, gallic acid, chebulic acid, corilagin and ellagic acid may constitute 30% of various phytochemicals [18, 19]. Though there are several studies on the anti-HSV-1 activity of *T. chebula* extract and its constituents namely chebulagic acid [15, 20, 21], but their activity against HSV-2 needs to be addressed.

Keeping in view of the above, the 50% ethanolic extract prepared from the fruits of *T. chebula* was evaluated for direct anti-viral activity and its impact on the attachment and penetration of the HSV-2 to the Vero cells. Chebulagic and chebulinic acids from *T. chebula* were also tested in these assays and their activity compared with well known drug, acyclovir. Additionally, the extract and pure compounds were also evaluated for in vitro anti-HSV-2 activity in post-infection plaque reduction assay.

## Methods

### Plant material, preparation of 50% ethanolic extract, and compounds

The fruits of *Terminalia chebula* were collected from the medicinal plants garden of Ayurveda Research Institute, Poojappura, Kerala and the identity was confirmed at Ayurveda Research Institute, Trivandrum, Kerala. The voucher specimen (HLL/04/2013) has been kept at Natural Products Division of HLL Lifecare Limited, Trivandrum, Kerala, India. Air and shade dried fruits were grinded and strained through a 30 mesh (0.5 mm). The finely grinded powder (24 g) was subjected to pressurized sequential extraction using Accelerated Solvent Extractor (ASE 150, Dionex Inc., Sunnyvale, CA, USA) essentially as described previously [22, 23]. In brief, the material was mixed with diatomaceous earth at 4:1 ratio. The mixture was placed into a sample cell (100 ml) and loaded onto the ASE 150 system. Extraction was performed using 50% ethanol under pressure (1500 psi) at 60 °C with a flush volume of 60% using 2 static cycles. The solvent was then evaporated in a rotary evaporator (Büchi Labortechnik AG, 9230 Flawil, Switzerland). The dried extract was weighed and used for further studies.

Highly pure commercial chebulagic acid and chebulinic acid, both purified from *T. chebula*, were obtained from MP Biomedicals, Ohio, Solon, USA. These compounds were used as reference standard to identify the corresponding peaks in 50% ethanolic extract prepared from fruits of *T. chebula* as well as to determine their anti-HSV-2 activity in various assays as described below. Both the compounds were dissolved in absolute ethanol (10 mg/ml) and further dilutions were made in the culture medium before use. Acyclovir (commercial name acycloguanosin) was purchased from Sigma-Aldrich Inc., St. Louis, MO, USA. It was dissolved in dimethyl sulphoxide (DMSO) to make stock solution of 7 mg/ml and further diluted in culture medium to prepare working concentration (s) prior to use. The final concentration of DMSO was < 0.1% in various assays where acyclovir was used.

### High performance liquid chromatography analysis

The Reverse Phase High Performance Liquid Chromatography (HPLC; LC Agilent, Agilent Technologies, Boblingen, Germany) of the plant extract was performed using a Reverse phase XTerra RP 18 column (4.6 × 250 mm, 5 μm; Waters Corporation, Milford, USA) and pure compounds (chebulagic and chebulinic acids) were used as reference standards. HPLC grade solvents were purchased from Merck, Mumbai, India. The solvent system used was 0.01% orthophosphoric acid in water : acetonitrile (80 : 20) with a flow rate of 1 ml/min. The peaks were detected at 272 nm. The extract was prepared at a concentration of 5 mg/ml and the standards at 1 mg/ml in the mobile phase solvent system and 20 μl of each was injected for analysis. The data was processed using open lab software (Agilent Technologies).

### Cells and viruses

African green monkey kidney cells (Vero cells) were obtained from National Centre of Cell Science, Pune, India and maintained in Dulbecco’s modified Eagle’s medium (DMEM; Sigma-Aldrich Inc.) supplemented with 10% fetal bovine serum (FBS; Biological Industries, Kibbutz beit HaemeK, Israel) and an antibiotic-antimycotic cocktail [Penicillin (100 units/ml), Streptomycin (100 μg/ml) and Amphotericin B (250 ng/ml); Pen-Strep-Ampho sol, Biological Industries]. For HSV-2 G strain (VR-734; ATCC, Rockville, USA) production, the Vero cell culture was maintained at 37 °C in a humidified atmosphere of 5% CO_2_. The HSV-2 strain was propagated in the 25-cm^2^ tissue culture flask for 72 h at 37 °C in CO_2_ incubator at multiplicity of infection (MOI) of 0.01 PFU/cell [24]. After three cycles of freezing/thawing, the supernatant was titrated on the basis of Plaque Forming Unit (PFU) as previously described [24] and stored in aliquots at −80 °C until use.

### Cytotoxicity assay

The cytotoxicity of the 50% ethanolic *T. chebula* extract, chebulagic acid, chebulinic acid and acyclovir was assessed by MTT [3-(4,5-dimethylthiazol-2-yl)-2,5-diphenyltetrazolium bromide; Sigma-Aldrich Inc.] assay [25]. In brief, Vero cells (1.25 × 10^4^/well) were seeded in 96-well cell culture plates (Greiner Bio-One, GmbH, Frickenhausen, Germany) and grown overnight at 37 °C in humidified atmosphere of 5% CO_2_. After 24 h, cells were treated with varying concentrations of the *T. chebula* extract, chebulagic acid, chebulinic acid and acyclovir in duplicate along with vehicle [dimethyl sulfoxide (DMSO) and/or ethanol] control for 48 h. Negative control included cells with medium only. After incubation, 20 μl of MTT (5 mg/ml in 50 mM PBS) was added per well and incubated for 4 h at 37 °C, followed by addition of MTT solvent (100 μl/well; 0.04 N HCl in absolute isopropanol). The absorbance (OD) was read at 540 nm with reference filter at 630 nm by using microplate spectrophotometer (ELX 800MS; BioTek Instrument Inc., Vermont, USA). Percent viability was calculated by dividing the OD obtained in treatment group by OD in the respective vehicle control multiplied by 100.

### Anti-HSV-2 activity of the extract of *T. chebula*, chebulagic acid and chebulinic acid

#### Direct anti-viral activity assay

To evaluate direct inactivation of HSV-2 by 50% ethanolic *T. chebula* extract, chebulagic acid and chebulinic acid, HSV-2 virus (100 PFU) was pre-incubated with sub-toxic serial concentrations of the *T. chebula* extract, chebulagic acid, chebulinic acid and acyclovir at 37 °C for 1 h. Subsequently, the contents were added to the confluent Vero cells monolayer growing in a 24-well cell culture plate (Corning Incorporated Costar, NY, USA) followed by incubation at 37 °C for 1 h under the humidified 5% CO_2_ atmosphere. After virus adsorption, the supernatant was aspirated and infected cells were washed twice with serum free DMEM and overlaid with 1% low melting point (LMP) agarose overlay medium. After 48 h incubation, cells were fixed with 10% formaldehyde (in 50 mM PBS), stained with 0.2% crystal violet and number of plaques counted. The percent plaque reduction was calculated as 100 – [(*P*_T_/*P*_C_) × 100], where *P*_T_ and *P*_C_ refer to the number of plaques in the presence and absence of the compound, respectively. The direct anti-viral activity was measured by plaque reduction as compared to the vehicle treated virus control. The minimal concentration of the compound required to inhibit 50% of plaque numbers (IC_50_) was calculated by regression analysis of the dose–response curves of the extract/compounds.

#### HSV-2 attachment and penetration assay

The attachment and penetration assays were performed essentially as described previously [26, 27] with slight modifications. In brief, for attachment assay, confluent Vero cells grown in 24-well cell culture plates were pre-chilled at 4 °C for 1 h. The medium was aspirated and virus (100 PFU/well) was added in the presence or absence of the varying concentrations of 50% ethanolic *T. chebula* extract, chebulagic acid, chebulinic acid and acyclovir to the Vero cells and incubated for 3 h at 4 °C. Subsequently, cells were washed twice with plain DMEM medium, and processed as described in direct anti-viral activity assay to determine IC_50_. In the penetration assay, HSV-2 virus (100 PFU/well) was added to the pre-chilled confluent monolayer of Vero cells growing in 24-well culture plate for 3 h at 4 °C to allow attachment. The medium was replaced with pre-warmed fresh medium containing varying concentrations of test extract/compounds and incubated at 37 °C to maximize virus penetration. After 30 min, the infected monolayer cultures were treated with warm PBS (pH 3.0) for 1 min to inactivate the non-penetrated viruses. After washing three times with serum-free medium, cells were overlaid with 1% LMP agarose and processed to quantitate the number of plaques after 48 h incubation as described above.

#### Post-infection virus plaque reduction assay

The anti-HSV-2 activity of 50% ethanolic *T. chebula* extract, chebulagic acid, chebulinic acid and acyclovir has been evaluated by using post-infection plaque reduction assay [28]. In brief, Vero cells (8 × 10^4^/well) were seeded in 24-well cell culture plate and incubated at 37 °C in a humidified atmosphere of 5% CO_2_ to form complete monolayer. After incubation, medium was removed and cells were infected with HSV-2 virus (100 PFU/well) for 1 h at 37 °C under humidified atmosphere of 5% CO_2_. After virus adsorption, viral inocula was subsequently removed by washing the cells twice with fresh serum free DMEM and cells were overlaid with 1% LMP agarose, containing varying concentrations (less than calculated CC_50_ values) of the 50% ethanolic extract from *T. chebula*, chebulagic acid and chebulinic acid. Acyclovir was used as positive control and agar overlay containing appropriate solvents (DMSO/ethanol), used to prepare test compounds comprises the vehicle control. After 48 h incubation, plates were processed and number of plaques counted.

### Statistical analysis

The values are expressed as mean ± standard error mean of three/four independent experiments performed in duplicate. For determination of the CC_50_ and IC_50_ values, nonlinear regression of concentration-response curves were prepared using GraphPad Prism 4 (GraphPad Software Inc., CA, USA). The statistical significance in reduction of plaques in presence of *T. chebula* extract, chebulagic acid, chebulinic acid and acyclovir versus respective vehicle treated virus control was determined by one-way ANOVA and *p* < 0.05 was considered statistically significant. Significance among various treatment groups was further analysed by using Tukey post-hoc test.

## Results

### *T. chebula* extract, chebulagic acid and chebulinic acid showed potent direct anti-viral activity

**Fig. 1:**
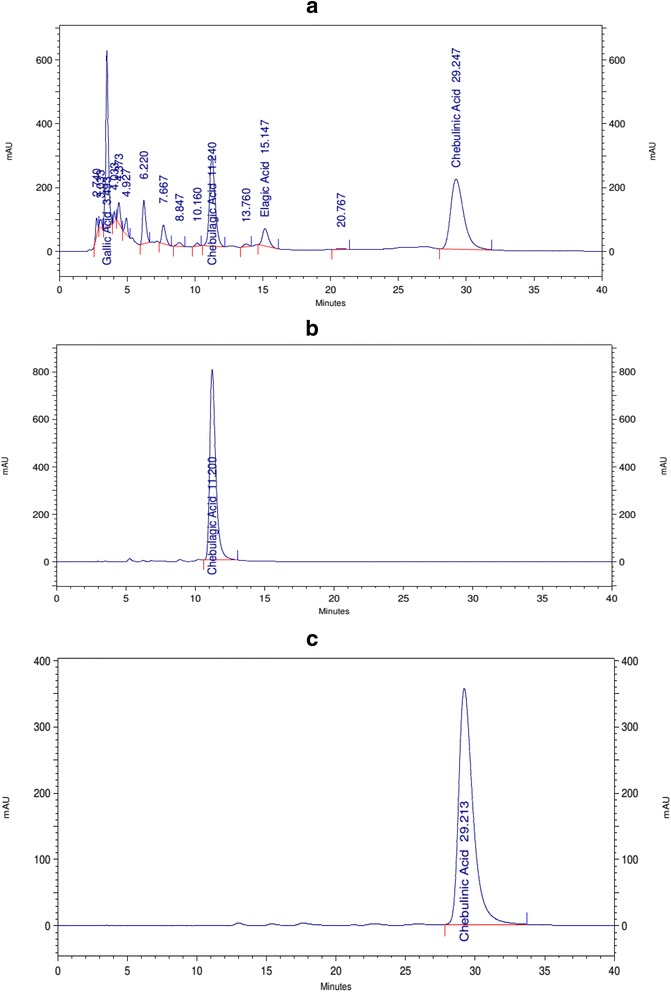
HPLC profiles of 50% ethanolic extract prepared from the fruits of *T. chebula*, chebulagic acid and chebulinic acid. The crude extract prepared from the fruits of *T. chebula* (100 μg), chebulagic acid (20 μg) and chebulinic acid (20 μg) were resolved by reverse phase HPLC using RP 18 column as described in Methods. The X-axis represents the time (min) and Y-axis represents the voltage (mAU) at 272 nm. Solvent used was acetonitrile : H_2_O (20 : 80 v/v) supplemented with 0.01% orthophosphoric acid. **a** 50% ethanolic extract prepared from the fruits of *T. chebula*, **b** chebulagic acid and (**c**) chebulinic acid. The peak corresponding to gallic acid in panel (**a**) was identified by running gallic acid standard (data not shown)

The 50% ethanolic extract from the fruits of *T. chebula* was prepared as described in Methods. From 24 g powder, the average yield of 50% ethanolic extract was 15 g (62.5% yield). Analysis of *T. chebula* extract by reverse phase HPLC revealed multiple peaks, furthermore chebulagic acid and chebulinic acid revealed a retention time of 11.2 and 29.2 min, respectively (Fig. 1). In addition to these two major peaks, peak with retention time of 3.4 min corresponded to gallic acid (Fig. 1).

Before evaluating the anti-HSV-2 activity of the 50% ethanolic extract prepared from the fruits of *T. chebula* or compounds chebulagic acid and chebulinic acid, their effect on the viability of Vero cells was determined by MTT assay as described in Methods. The extract of *T. chebula* revealed 409.71 ± 47.70 μg/ml CC_50_ value (Table 1). The chebulagic and chebulinic acids showed > 95% cell viability when tested up to 200 μg/ml. The CC_50_ value of 351.85 ± 48.80 μg/ml was observed with acyclovir (Table 1).

**Table 1:** Cytotoxicity and direct antiviral activity against HSV-2 of the 50% ethanolic extract prepared from fruits of *T. chebula*, chebulagic acid, chebulinic acids and acyclovir in Vero cells

Test compounds	^a^CC_50_ (μg/ml)	^b^IC_50_ (μg/ml)
*T. chebula* 50% ethanolic extract	409.71 ± 47.70	0.01 ± 0.0002
Chebulagic acid	>200^c^	1.41 ± 0.51
Chebulinic acid	>200^c^	0.06 ± 0.002
Acyclovir	351.85 ± 48.80	29.04 ± 1.04

Values in this table represent the mean ± SE of four independent experiments performed in duplicate

^a^CC_50_ was the concentration that showed viability of 50% of the Vero cells

^b^IC_50_ was the concentration that inhibited 50% of HSV-2 activity in direct anti- viral activity assay using Vero cells as host

^c^ > 95% cells were viable at 200 μg/ml

Anti-HSV-2 activity by direct inactivation of the virus was tested in the presence of crude extract/compounds. HSV-2 virus was pre-treated with varying concentrations of *T. chebula* extract, chebulagic acid, chebulinic acid and acyclovir followed by analysis of their ability to infect Vero cells by formation of plaques as described in Methods. The pre-treatment of HSV-2 with the plant extract, chebulagic acid as well as chebulinic acid showed a significant reduction in the formation of plaques as compared to vehicle treated virus control group with IC_50_ values of 0.01 ± 0.0002, 1.41 ± 0.51, and 0.06 ± 0.002 μg/ml respectively (Table 1). The differential anti-HSV-2 activity of the above treatment groups with respect to each other was not significant using one way ANOVA. The direct antiviral activity of the *T. chebula* extract, chebulagic acid and chebulinic acid was approximately 20 to 2900 fold higher than acyclovir (IC_50_ = 29.04 ± 1.04 μg/ml; Table 1).

### *T. chebula* extract, chebulagic acid and chebulinic acid effectively inhibited HSV-2 attachment and penetration to the Vero cells

**Fig. 2:**
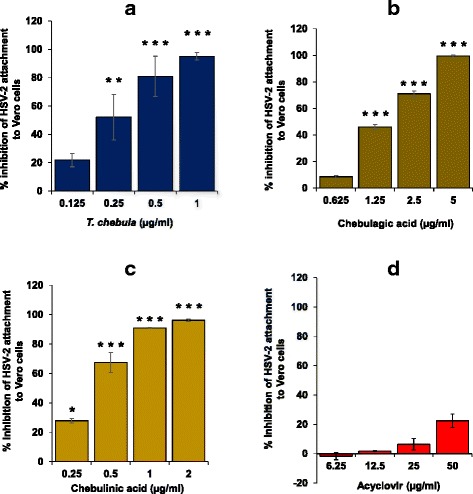
Efficacy of the *T. chebula* extract, chebulagic acid, chebulinic acid and acyclovir to inhibit attachment of HSV-2 to Vero cells. HSV-2 (100 PFU) virus along with various concentrations of the *T. chebula* extract, chebulagic acid, chebulinic acid and acyclovir was incubated for 3 h with the pre-chilled Vero cells seeded in 24-well plates at 4 °C and processed for inhibition in the attachment of the virus as described in Methods. The Y-axis represents percent inhibition in the number of plaques with respect to the vehicle treated virus control group. Values are expressed as mean ± SEM of the three independent experiments performed in duplicate. Various panels are (**a**) extract of *T. chebula,* **b** chebulagic acid, **c** chebulinic acid and (**d**) acyclovir. ^*^ *p* ≤ 0.05, ^**^ *p* ≤ 0.01, and ^***^ *p* ≤ 0.001 between treated and vehicle control at respective concentration of the extract/compound

Further, the effect of *T. chebula* extract, chebulagic acid, chebulinic acid and acyclovir was also evaluated on the attachment and penetration of HSV-2 to the Vero cells as described in Methods. *T. chebula* extract, chebulagic acid and chebulinic acid significantly inhibited in vitro binding of HSV-2 to Vero cells in a dose-dependent manner with the IC_50_ values of 0.48 ± 0.20, 1.66 ± 0.30, and 0.29 ± 0.01 μg/ml as compared to vehicle treated virus control respectively (Fig. 2). The differences among the treatment groups of plant extract, chebulagic acid and chebulinic acid for inhibition of HSV-2 attachment to Vero cells was significant by one way ANOVA analysis. Furthermore, analysis by Tukey post-hoc test revealed that anti-HSV-2 activity in the attachment assay by chebulinic acid was significantly higher than chebulagic acid. Acyclovir, when tested up to a concentration of 50 μg/ml did not show any significant (*p* > 0.05) inhibition in the HSV-2 binding to Vero cells as revealed by the number of plaques observed as compared to DMSO treated negative control (Fig. 2). Further, pre-treatment of the Vero cells with the 10 μg/ml of *T. chebula* extract, chebulagic acid & chebulinic acid and 1 μg/ml of acyclovir followed by infection with HSV-2 did not show any significant reduction in the number of viral plaques as compared to vehicle treated cells, suggesting that the above extract/compounds do not have any inhibitory effect on the host cell receptors (data not shown).

**Fig. 3:**
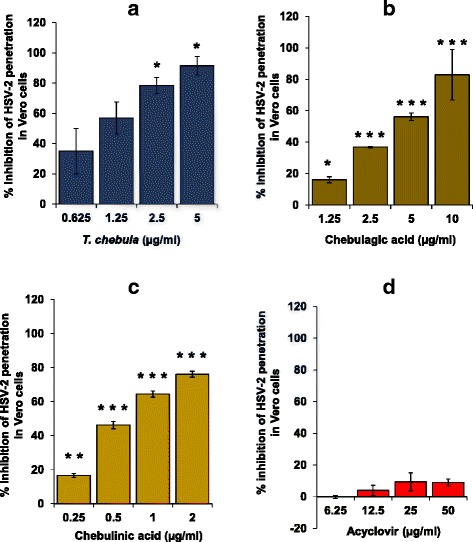
Efficacy of the *T. chebula* extract, chebulagic acid, chebulinic acid and acyclovir to inhibit penetration of HSV-2 to Vero cells. Pre-chilled confluent monolayer of Vero cells growing in 24-well culture plate were incubated with HSV-2 (100 PFU) for 3 h at 4 °C followed by incubation with the varying concentrations of the extract of *T. chebula* (**a**), chebulagic acid (**b**), chebulinic acid (**c**) and acyclovir (**d**) at 37 °C for 30 min to allow virus penetration followed by assessment of plaque formation as described in Methods. Values are expressed as percent inhibition in the number of plaques as compared to vehicle treated virus control. Each bar represents the mean ± SEM of three independent experiments performed in duplicate. ^*^ *p* ≤ 0.05, ^**^ *p* ≤ 0.01, and ^***^ *p* ≤ 0.001 between treated and vehicle control at respective concentration of the extract/compound

In addition to the attachment assay, we also observed a concentration dependent significant inhibition of HSV-2 penetration to the Vero cells by the *T. chebula* extract, chebulagic acid and chebulinic acid with the IC_50_ values of 0.99 ± 0.52 μg/ml, 4.65 ± 0.02 μg/ml, and 0.68 ± 0.05 μg/ml respectively with respect to vehicle treated virus control (Fig. 3). The differences among the treatment groups of plant extract, chebulagic acid and chebulinic acid for inhibition of HSV-2 penetration in the Vero cells was significant by one way ANOVA analysis. Further analysis of the data by Tukey post-hoc test revealed significant differences between chebulagic acid treatment group with respect to plant extract or chebulinic acid treatment groups. However, no significant difference was observed between plant extract versus chebulinic acid treatment group. As in attachment assay, chebulinic acid in the penetration assay also showed highest anti-HSV-2 activity. Acyclovir (50 μg/ml) did not reveal any significant (*p* > 0.05) decrease in HSV-2 penetration to the Vero cells under similar experimental conditions (Fig. 3).

### *T. chebula* extract, chebulagic acid and chebulinic acid showed reduced anti-HSV-2 activity as compared to acyclovir in post-infection plaque reduction assay

**Fig. 4:**
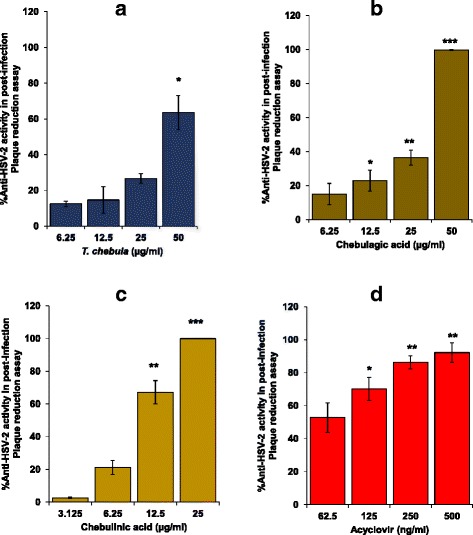
Anti-HSV-2 activity of the *T. chebula* extract, chebulagic acid, chebulinic acid and acyclovir in post-infection plaque reduction assay. Vero cells growing as monolayer in 24-well culture plate were infected with HSV-2 (100 PFU/well) followed by 1% low melting point agarose overlay medium containing varying concentration of the 50% ethanolic extract of *T. chebula*, chebulagic acid, chebulinic acid and acyclovir. After 48 h incubation, plates were processed to assess the number of plaques as described in Methods. The Y-axis represents percent inhibition in the number of plaques with respect to the vehicle treated virus control group and X-axis the concentration of the test extract/compounds. Each bar represents mean ± SEM of the three independent experiments performed in duplicate. Various panels are **a** extract of *T. chebula,* **b** chebulagic acid, **c** chebulinic acid and **d** acyclovir. ^*^ *p* ≤ 0.05, ^**^ *p* ≤ 0.01, and ^***^ *p* ≤ 0.001 respectively between treated and vehicle control at tested concentration of the extract/compound

In the post-infection plaque reduction assay, acyclovir showed dose dependent significant inhibition of HSV-2 replication with an IC_50_ value of 71.80 ± 19.95 ng/ml (Fig. 4). However, extract (IC_50_ = 50.06 ± 6.12 μg/ml) as well as chebulagic (IC_50_ = 31.84 ± 2.64 μg/ml) and chebulinic (IC_50_ = 8.69 ± 2.09 μg/ml) acids showed approximately 120–700 fold lower IC_50_ values as compared to acyclovir in the post-infection plaque reduction assay (Fig. 4). Using Tukey post-hoc test, chebulinic acid showed significantly higher anti-HSV-2 activity as compared to plant extract as well as chebulagic acid. However, anti-HSV-2 activity was not significantly different between plant extract versus chebulagic acid.

## Discussion

In the present study, we have shown that the 50% ethanolic extract prepared from the fruits of *T. chebula* has a very potent direct antiviral activity against HSV-2 (Table 1). The direct anti-viral activity of the aqueous extract prepared from the seeds of *T. chebula* on influenza A (H3N8) virus has also been reported [16]. Casuarinin, a hydrolyzable tannin isolated from the bark of *Terminalia arjuna* Linn also showed potent virucidal activity against HSV-2 at a concentration of 25 μM [29]. The observed direct anti-viral activity by the two hydrolyzable tannins namely chebulagic acid and chebulinic acid purified from *T. chebula* against HSV-2 is reported for the first time, which seems to be higher than casuarinin. The direct anti-viral activity of *T. chebula* extract, chebulagic acid and chebulinic acid was also significantly (*p* < 0.05) higher than acyclovir, a well known drug used for the treatment of HSV infection. We speculate that the direct anti-viral activity of *T. chebula* extract, chebulagic acid and chebulinic acid may be due to their interaction with viral surface glycoproteins, causing alteration in its function or by making virus particles inert and prevent their attachment and spreading to the Vero cells. Indeed hydrolyzable tannins such as chebulagic acid and punicalagin have been shown to bind to glycoproteins of various viruses thereby inhibiting their entry to the host cells as well as prevent cell-to-cell spread (20, 21).

HSV requires its glycoproteins (gB, gC, gD, gH and gL) to interact with the host cell receptors (heparan sulphate, nectin-1 and nectin-2) followed by penetrating the membrane by fusion process [30, 31]. *T. chebula* extract, chebulagic acid and chebulinic acid showed potent inhibition of HSV-2 attachment (Fig. 2) as well as penetration (Fig. 3) to Vero cells. It has been shown that casuarinin, inhibited the attachment and penetration of HSV-2 to Vero cells broadly through the disturbances of the viral glycoproteins [29]. Chebulagic acid and punicalagin, the hydrolyzable tannins isolated from the fruits of *T. chebula*, inhibited HSV-1 viral particles and prevented their binding, penetration and cell-to-cell spread, as well as secondary infection [20]. However, pre-treatment of the host cells with chebulagic acid and punicalagin were not effective in prevention of HSV-1 infection. Their inhibitory activities targeted HSV-1 glycoproteins involved in the attachment and membrane fusion, by blocking interaction with host cell surface glycosaminoglycans. Antiviral activity of chebulagic acid and punicalagin was significantly reduced in mutant cell lines deficient for heparan sulfate and chondroitin sulfate suggesting that these tannins may be interfering in the interaction of HSV-1 glycoproteins with heparan sulfate/chondroitin sulfate rather than lysis of viral membrane [20]. It is likely that the *T. chebula* extract, chebulagic acid and chebulinic acid also target HSV-2 glycoproteins that are responsible for its binding to host cell receptors. Failure of acyclovir to show any significant decrease in the attachment as well as penetration of HSV-2 to the host cells may be due to the fact that it primarily works intra-cellularly by inhibiting virus replication.

The results shown in the present study revealed that 50% ethanolic extract prepared from the fruits of *T. chebula*, chebulagic acid and chebulinic acid also inhibited post-fusion HSV-2 replication or spread to other cells, which was approximately 120–700 fold lower as compared to acyclovir. Keeping in view of the large molecular weight of chebulagic acid and chebulinic acid, it is likely that they may not penetrate the Vero cells and hence may not interfere in intracellular virus replication. In contrast, acyclovir will enter the cells and inhibit HSV-2 replication. Acyclovir is converted in the virus infected cells to acyclovir monophosphate using viral-encoded thymidine kinase and subsequently to acyclovir triphosphate by using host cell enzymes, which prevent virus DNA synthesis and thus replication by inhibiting the viral DNA polymerase [32]. It is likely that *T. chebula* extract, chebulagic acid and chebulinic acid binds to viral glycoproteins expressed on the infected Vero cell surface thereby rendering their unavailability for cell-to-cell spread. However, further studies will be required to understand the plausible mechanism by which *T. chebula* extract, chebulagic acid and chebulinic acid exhibit anti-HSV-2 activity in the post-infection plaque reduction assay.

*T. chebula* fruit is one of the important ingredients of Triphala-an important herbal preparation used for centuries in the traditional Indian system of Ayurveda as colon cleanser, digestive, diuretic and laxative [33]. The extract prepared from the fruits of *T. chebula* or its constituents have shown diverse pharmacological activities which include, antioxidant and free radical scavenging activity, anticarcinogenic activity, hepatoprotective activity, cardioprotective activity, cytoprotective activity, antidiabetic activity, antibacterial, fungal and viral activity, gasterointestinal motility improving and anti-ulcerogenic activity etc. [17, 34–38]. Keeping in view of its acetylcholinestrase inhibitors activity, antioxidant and anti-inflammatory effects, its potential for the treatment of alzheimer’s disease is also being explored [39]. Interestingly, in vivo anti-viral property of chebulagic acid, was also demonstrated by treating mice challenged with a lethal dose of human enterovirus 71 (major causative agent of hand, foot and mouth disease in younger children), which led to reduce mortality and clinical symptoms mediated by inhibiting viral replication [40]. In another study, the extract prepared from *T. chebula* Retz when administered along with acyclovir significantly curtailed the development of skin lesion and prolonged the mean survival rate of HSV-1 infected mice as compared to when administered either acyclovir or herbal extract alone [15]. These studies suggest the enormous clinical potential of *T. chebula* for a variety of ailments. The obvious question comes to mind about the safety of the *T. chebula*-based herbal preparations. The ethanolic extract of *T. chebula* fruits did not show any mutagenic properties as tested in bacterial mutation assays and also did not reveal any toxic effects when repeatedly administered orally in rats [41]. The anti-mutagenic activity of the hydrolyzable tannins purified from *T. chebula* has also been shown by another independent study [42]. The fruits from *T. chebula* could reduce the lead and almunium induced genotoxicity [38]. These studies suggest that *T. chebula* extract and hydrolyzable tannins are safe molecules to be used as therapeutic agents against HSV. In contrast, a known side effect of prolonged treatment with acyclovir is nephrotoxicity [43]. However, the extract of *T. chebula* has nephroprotective activity [44]. Use of *T. chebula* extract, chebulagic and chebulinic acids could improve the prognosis of sexually transmitted HSV-2 as well as reduction of sexually transmitted HIV-1 as co-infection. The extract of *T. chebula* will also be useful against viruses that use glycosaminoglycans as entry door in to the host such as Hepatitis C, HIV and dengue viruses. However, extensive investigations are needed to exploit its therapeutic potential for drug resistant viruses.

## Conclusions

To the best of our knowledge, we have demonstrated for the first time, anti-HSV-2 activity of *T. chebula*. Due to potent direct antiviral activity against HSV-2 including its attachment as well as penetration by *T. chebula* extract, chebulagic acid and chebulinic acid, which is higher than acyclovir, this plant may be a good source to identify natural product (s). However, additional studies will be required to determine the mechanism by which *T. chebula* and its constituents exhibit anti-HSV-2 activity.

## Abbreviations

ASE: Accelerated solvent extractor
CC_50_: Concentration that shows 50% of cytotoxic effect
CMV: Cytomegalovirus
DMEM: Dulbecco’s modified Eagle’s medium
DMSO: Dimethyl sulfoxide
FBS: Fetal bovine serum
HIV: Human immunodeficiency virus
HPLC: High Performance Liquid Chromatography
HSV: Herpes simplex virus
IC_50_: Inhibitory concentration producing 50% reduction in plaque formation
LMP: Low melting point
MOI: Multiplicity of infection
MTT: 3-(4,5-dimethylthiazole-2-yl)-2-5-diphenyltetrazolium bromide
PFU: Plaque forming unit
STI: Sexually transmitted infection
